# The effects of the European e-cigarette health warnings and comparative health messages on non-smokers’ and smokers’ risk perceptions and behavioural intentions

**DOI:** 10.1186/s12889-018-6161-7

**Published:** 2018-11-14

**Authors:** Catherine Kimber, Daniel Frings, Sharon Cox, Ian Albery, Lynne Dawkins

**Affiliations:** Centre for Addictive Behaviours Research, Division of Psychology, School of Applied Sciences, 103 Borough road, London South Bank University, London, SE1 0AA UK

**Keywords:** Electronic cigarettes, E-cigarettes, Warning labels, Tobacco products directive, Health messages, Risk perceptions, Quit intentions, Motivation to quit

## Abstract

**Background:**

Article 20 of the EU Tobacco Products Directive [TPD] stipulates that e-cigarette packets and refill products must carry a nicotine addiction health warning. Although previous studies conducted in North America have found that perceived harm, addictiveness and intention to use declined following exposure to e-cigarette health warnings, possible effects of the TPD health warnings on smokers and non-smokers has not been studied. This study will investigate the effects of the EU TPD e-cigarette health warnings and a comparative harm message (COMP; developed specifically for this study) on smokers’ and non-smokers’ perceptions of harm, addictiveness and social acceptability of e-cigarettes. Additionally, the potential effects of the TPD warnings and the COMP on smokers’ intentions to purchase and use e-cigarettes will be explored.

**Methods/design:**

A sample of 2400 UK residents will be recruited in this experimental, randomised design, with Smoking status (Smoker vs. Non-smoker), TPD presence (TPD1 vs. TPD2 vs. No-TPD) and COMP presence (Presence vs. Absence) as between subjects independent variables, and Time (pre-post exposure of images) as a within subjects factor. Dependent variables comprise self-reported perceived harm, addictiveness, social acceptability, e-cigarettes’ effectiveness, intentions to purchase and use e-cigarettes. Cigarette dependence, previous e-cigarette exposure, and baseline intentions to quit will be measured as covariates.

**Discussion:**

Health warnings, such as those implemented by the TPD, may help to prevent non-smokers from e-cigarettes use, but it is possible that they may inadvertently deter smokers from initiating use and substituting their tobacco smoking for e-cigarettes use if their content is deemed too negative. It is hoped that this study will help identify the most effective message or combination of messages that encourage use among smokers without promoting use among non-smokers.

**Trial registration:**

ISRCTN registry ISRCTN76967031; date of registration: 23/10/18.

## Background

Health warnings on cigarette packets can be an effective tool to increase awareness about the dangers of smoking. Research suggests that they can act as a deterrent to smoking and promote cessation [1, 2]. Consequently, as part of a comprehensive tobacco control strategy, tobacco warning labels have become central to anti-smoking education campaigns worldwide. In addition to reducing smokers’ desire for tobacco cigarettes [3, 4], warning labels have the potential to prevent initiation in non-smokers [5]. A systematic review of 20 countries reported that strengthening health warnings on cigarette packs was associated with increased knowledge of the harms of smoking, a reduction in smoking consumption, increased quit attempts and reduced smoking prevalence [2]. Novel, larger, graphic health warnings that convey a loss-framed message (focus on harms of smoking rather than gains from quitting) and lack of branding (standardised packaging), have been shown to increase attention to the message and improve perceptions of health risks and quitting-related behaviour [6–11].

E-cigarettes are a potential tobacco harm reduction product estimated to carry approximately 5% of the health risk of tobacco smoking [12–14]. Since their introduction, uptake and awareness has increased dramatically [15, 16]. An estimated 2.9 million adults in Great Britain currently use e-cigarettes [15, 16] over half (52%) of e-cigarette users are now former smokers and 45% are continuing smokers (dual users) [16]. Smoking cessation and reduction remain the most commonly cited reasons for use [16, 17]. Though some reports suggest otherwise [18], there is increasing evidence for their effectiveness for smoking cessation [19, 20].

Although not completely risk free, growing evidence now suggests they are considerably less harmful than tobacco cigarettes [21–28]. Owing to this reduced risk profile, health and policy-makers (in the UK) consensually recommend that smokers who are unwilling or unable to quit should be encouraged to switch to e-cigarettes [12–14]. Worryingly, in recent years, public misperceptions of harm associated with e-cigarettes use have increased, with only 13% of survey respondents in the UK correctly believing that e-cigarettes are considerably less harmful than tobacco smoking [16]. In a sample of 4058 Greek residents, only 5% perceived e-cigarettes as less harmful than cigarettes in 2017 [29]. Similarly, in the US, between 2012 and 2015, the odds of perceiving e-cigarettes to be equally or more harmful than smoking has tripled [30].

The need to communicate the reduced risk status of e-cigarettes compared with tobacco cigarettes has been expressed before [31] and including relative risk messages on e-cigarette packages may be one way to communicate this. Nevertheless, current messages on e-cigarette packaging present an addiction health warning which may reinforce negative beliefs and reduce smoking cessation attempts using e-cigarettes.

Article 20 of the EU Tobacco Products Directive [TPD] [32] stipulates that e-cigarette packets and refill products must carry a health warning covering 30% of the packaging, either: i) ‘This product contains nicotine which is a highly addictive substance’, or ii) ‘This product contains nicotine which is a highly addictive substance. It is not recommended for use by non-smokers’. Aside from a recent study which suggests that addiction warnings appearing on hardware items (e.g. atomisers) lead to confusion amongst vapers [33], the impact of these health warnings on e-cigarette risk perceptions, smoking cessation and e-cigarette purchasing intentions in smokers has received very little attention. To date, research on e-cigarette health warnings has been concentrated in the US, Canada and Hong Kong. Two experimental studies have found an increase in harm and addictiveness perceptions and decrease in intention to use in US and Canadian young non-smokers following exposure to e-cigarette health warnings which conveyed the potential health and addiction risks of e-cigarettes [34, 35]. Whilst reducing e-cigarette appeal among non-smokers is clearly desirable, findings from a recent focus group with e-cigarette users and smokers suggested that health warnings that are deemed too negative may have the unintended consequence of reducing appeal among smokers who may be considering e-cigarettes for smoking cessation [31]. E-cigarette advertisements that include an addiction warning have been shown to increase health-risk beliefs in smokers and e-cigarette users, which in turn, negatively influence willingness to try the product [36]. Conversely, advertising messages that focused on differences between cigarettes and e-cigarettes (e.g. healthier, helps to quit smoking) rather than similarities (feels like smoking, relieves cravings) created more interest among smokers in trying an e-cigarette [37].

Given that comparative health messages (emphasising the reduced risk status of e-cigarettes compared with tobacco smoking) may increase the perceived utility and the value of using e-cigarettes as a quit aid (by contextualising warnings of addictiveness and the relative toxicity profile of e-cigarettes against tobacco cigarettes) they should, from an expectancy-value perspective, motivate positive health choices. Numerous approaches have suggested that *behavioural enactment* is predicated on the formation of a relevant *intention* which in turn are influenced by judgments of risk or expectancies of experiencing related outcomes (*risk perceptions*) [38, 39]. Such associations are well documented in tobacco products research. Whilst *intentions to trial* has been found to be a strong predictor of *future use* [40], other work has shown an association between *susceptibility to use* (*intentions*), *normative beliefs* (the perception that the target behaviour is socially acceptable) and *risk-based beliefs* (addiction and harm) [41]. Given this, it is likely that *exposure to advertisement materials* (i.e. health related warning labels and messages, which are likely to affect *intentions, normative* and *risk-based beliefs*) can influence *purchasing behaviours* by shaping relevant *perceptions* [42, 43]. In the current work, exposure to various warning labels will be manipulated and relevant *perceptions* measured to explore if, and to what extent, TPD warnings exert an effect on perceptions of harms, addictiveness and effectiveness of e-cigarettes and whether e-cigarettes are perceived to be socially acceptable. We will also test the effects of a comparative health message (generated prior to commencing this work and described below) alone and in conjunction with the TPD. Depending on the pilot study results, this comparative message will either convey the potential gains associated with switching away from tobacco smoking (e.g. ‘*Using an e-cigarette doubles your chances of quitting smoking’*), or will be framed to highlight gains associated with avoidance of the harmful health consequences of maintaining smoking (e.g. ‘*Completely switching to e-cigarettes lowers your risk of smoking related diseases’*).

### Objectives

This Cancer Research UK (CRUK) funded study will investigate the effects of the TPD e-cigarette health warnings and a comparative harm message on smokers’ and non-smokers’ perceptions of harm, addictiveness and social acceptability of e-cigarettes. Additional aims are to evidence the potential effects of the TPD warnings, as they are implemented, on smokers’ intentions to purchase and use e-cigarettes in future quit attempts. Lastly, the potential benefits of providing a comparative harm message either in addition to the TPD warning or as a stand-alone message will be explored.

### Hypotheses

- H_1_: Participants exposed to the TPD health warning will rate e-cigarettes as more harmful and addictive compared to those exposed to i) the TPD warning combined with a comparative message (TPD+), ii) the comparative health message (COMP) alone and iii) the no message control condition.
- H_2_: For participants exposed to the TPD health warnings, post-exposure scores on intentions to purchase and use e-cigarettes will decrease compared to those exposed to i) the COMP alone, ii) the TPD+ messages, or iii) no message (control).
- H_3_: TPD health warnings will decrease quit intentions in smokers and the comparative health message (COMP and TPD+ conditions) will attenuate this effect.
- For H_1_ and H_2_ we will explore differences between smokers and non-smokers.

## Method

### Design

An experimental, randomised design will be used, with Smoking status (Smoker vs. Non-smoker), TPD presence (TPD1 vs. TPD2 vs. No-TPD; see below) and COMP presence (Presence vs. Absence) as between subjects independent variables, and Time (pre-post exposure of images/health messages) as a within subjects factor. Dependent variables comprise self-reported perceived harm, addictiveness, social acceptability, effectiveness, intentions to purchase and use of e-cigarettes. Smoking dependence, previous e-cigarette exposure, and baseline intentions to quit will be measured as covariates. For the E-cigarette Health Message factor, participants will be exposed to one of the six stimuli versions (see below). Messages will be displayed on e-cigarette packages as per the current EU-TPD warning labels on e-cigarette products. Warning label stimuli are:

- TPD1: TPD health warning as per currently implemented in the UK. “*This product contains nicotine which is a highly addictive substance*”
- TPD2: TPD longer health warning as currently implemented in many EU countries: “*This product contains nicotine which is a highly addictive substance. It is not recommended for non-smokers*”
- COMP: Comparative health message as generated in the pilot study (using the same parameters used for the TPD warning labels; font, font colour, size and placement on the pack)
- TPD1+: The TPD health warning (TPD1) in combination with the comparative message (using the same parameters above)
- TPD2+: The TPD longer health warning (TPD2) in combination with the comparative message (using the same parameters as above).
- No message: A no message condition using the same e-cigarette packages

The primary outcome variables for this study will be smokers’ and non-smokers’ perceptions of e-cigarettes associated with i) harm, ii) addictiveness, iii) social acceptability, iv) effectiveness, v) intentions to purchase, and vi) intentions to use e-cigarettes. Key outcome variables specifically for smokers will be intentions (i) to quit and (ii) use e-cigarettes in a quit attempt. All measures will be taken before and after exposure to messages.

### Study population/ sample size

The sample will include 2400 participants (*N* = 1200 smokers; N = 1200 non-smokers) all residents in the UK. Given an effect size of between OR = 0.41 and 0.52 (the effect sizes observed in intent to purchase e-cigarettes between control, ingredient and industry warnings respectively) [44] and converting the OR to effect size F using Chinn [45] methodology (Fs = 0.41–0.52) suggest the current study design will need to be powered to detect medium to large effects. The proposed sample (*N* = 1200) allows for detection of small (F = 0.12) main effects and interactions.

Smokers will be matched to the target population of smokers’ demographics in the general population using ONS data by deriving best estimates of required numbers in each condition required at levels of age (banded into two segments), gender (two segments) and Socio-Economic Status (SES) (four segments). The latter will be derived from the number of participants required in the four SES segments (managerial and professional occupations; intermediate occupations; routine and manual occupations; never worked or unemployed). Thereafter, the number in each of these bands for gender and then age bands will be derived resulting in a target number of each of the 16 combinations. A panel agency (Market Research Focus Group Recruiter (MRFGR, a division of AGENTC Ltd)) will be contracted to recruit and randomise to each of these targets using a set of block lists provided by the research team. The same stratification levels will be used for non-smokers matched to population level statistics.

Block randomisation will be used for allocation of conditions and demographic variables (including smoking status) will be stratified so that the sample of smokers is representative of the target population in line with ONS data. Each participant will be assigned to one of the six conditions to obtain equal group sizes using IBM SPSS.

### Recruitment

Participants will be recruited via MRFGR. All payment of participants will be handled by the panel agency. Participants are awarded with points which they receive after completing the survey. The number of points depends on the length of study and can be redeemed in cash or vouchers (i.e. Amazon). Respondents can redeem their cash after cumulating 150 points or more (equating to £15).

### Inclusion/exclusion criteria

Smokers will be matched to the target population of smokers as described above. Inclusion criteria are: adult aged 18+, resident in the UK, fluent in English. Exclusion criteria are: under 18 years of age, resident outside of the UK, exclusive vapers, dual users (i.e. concurrent use of e-cigarettes and any tobacco consumption), former smokers, and non-fluent in English. Inclusion and exclusion criteria will be communicated to participants before they provide consent.

### Measures

#### Baseline measures

Demographic variables will include gender, age, ethnicity, occupation and economic activity and highest attained qualification in line with data collected by the Office for National Statistics (ONS). Occupation will be measured with a single item questionnaire using four categories ‘routine and manual’, ‘intermediate’, ‘managerial and professional occupation, ‘never worked & long-term unemployed’ [46].

Smoking status will be classified as ‘never smoked’ (individuals who have smoked fewer than 100 cigarettes in their life time and have not smoked in the past 30 days), ‘daily smoker’ (anyone who has smoked daily for the past 30 days and has smoked more than 100 cigarettes in their life time), ‘non-daily, occasional and social smoker’ (individuals who do not smoke every day but, for example, might smoke once a week providing they have smoked more than 100 cigarettes in their life time and in the past 30 days) based on the criteria European risk monitoring project [35, 47, 48].

For smokers, motivation to quit will be measured using the validated single-item instrument, Motivation to Stop Scale (MTSS), which measures intention, desire to stop smoking and the immediacy of their intended quit date (e.g. “I don’t want to stop smoking, I REALLY want to stop smoking but I don’t know when I will, I REALLY want to stop smoking and intend to in the next 3 months, I REALLY want to stop smoking and intend to in the next month”) [49]. Cigarette dependence will be measured using the Fagerström Test for Cigarette Dependence (FTCD) (e.g. the number of cigarettes smoked per day, time to first cigarette of the day, etc.) [50].

### Stimuli development

#### Comparative health message formulation

A pilot study will be conducted to generate the most appropriate comparative health message from a list of eight messages (which were selected from an initial list of 26 on the basis of receiving the highest ratings previously from seven experts in the field). This will provide a robust comparative health message that has been evaluated for accuracy, persuasiveness and clarity by a panel of experts before being tested in a pilot study of 1000 participants (non-smokers and smokers) on comprehensibility, credibility and convincingness. The chosen health message will be displayed on various e-cigarette packages in line with the current EU-TPD requirements to ensure consistency (i.e. occupy 30% of the surface of the pack, printed in black Helvetica bold type on a white background) as described above.

#### Outcome variables

Prior to, and following the experimental exposure to one of the e-cigarette health messages described above (each presented for a standardised period of 30 s), perceptions of e-cigarettes and intentions will be measured on the following scales: i) harmfulness, ii) addictiveness and ii) socially acceptable, iv) effectiveness as a cessation aid, v) intention to use, vi) intention to purchase and, for smokers only, vii) intentions to quit and use e-cigarettes in a future quit attempt (all on a 7-point rating scales with the anchors “Extremely, to Not at all” scored from 7 to 1; see Table 1 for the full list of constructs). In order to minimise response bias, unrelated (filler) questions will be presented following exposure of the message stimuli in addition to the aforementioned outcome measures. Examples of such questions will include the following: “Which e-cigarette did you find the most appealing?”; “Which e-cigarette (if any) would you be most likely to use?”; “Which e-cigarette did you think looked most like a cigarette?”. Participants will also be asked: “Did you notice a health message on the e-cigarette pack?”; “Did you think the health message was positive, negative, neutral?”

**Table 1:** List of constructs (outcome measures)

Constructs	Statements	Anchors
Perceptions of e-cigarettes	How harmful do you think e-cigarettes are?	☐ Extremely (…) ☐ Not at all
	How addictive do you think e-cigarettes are?	☐ Extremely (…) ☐ Not at all
	How socially acceptable do you think e-cigarettes are?	☐ Extremely (…) ☐ Not at all
	How effective do you believe e-cigarettes to be as a quit aid	☐ Extremely (…) ☐ Not at all
Intentions	How likely are you to use an e-cigarette	
	a) in the next month b) in the next 6 months	☐ Extremely (…) ☐ Not at all ☐ Extremely (…) ☐ Not at all
	How likely are you to purchase an e-cigarette	
	a) in the next month b) in the next 6 months	☐ Extremely (…) ☐ Not at all ☐ Extremely (…) ☐ Not at all
	For smokers only	
	How likely is it that you will make a quit attempt:	
	a) in the next month b) in the next 6 months	☐ Extremely (…) ☐ Not at all ☐ Extremely (…) ☐ Not at all
	How likely are you to use an e-cigarette in a quit attempt	
	a) in the next month b) in the next 6 months	☐ Extremely (…) ☐ Not at all ☐ Extremely (…) ☐ Not at all

Note: Each of these statements will be presented prior to and following exposure to one of the e-cigarette health messages

### Study setting/procedure

Participants will be contacted by the panel agency and provided with a link. On clicking the link they will be presented with an online information sheet and then prompted to provide consent. Participants will be asked to rate the images on the constructs highlighted in Table 1 (i.e. all items measuring perceptions and intentions) before and after presentation of the allocated health message displayed on a series of different e-cigarette images. Thereafter, they will be presented with the non-related filler questions, before completing a set of questionnaires to collect demographic information such as age, gender, ethnicity, occupation, SES, highest qualification, smoking status and past e-cigarette use. The FTCD [50] and MTSS questionnaires [49] will be administered to smokers only, to measure cigarette dependence and motivation. Lastly, participants will be presented with an on-screen debriefing information letter.

### Data management and planned dissemination

The study will use a panel agency that is GDPR (General Data Protection Regulations) compliant. All data will be kept in accordance with the study protocol and with GDPR’s requirements under password protected networked PCs and any documents on hard copies in locked filing cabinets in a locked office. Once finalised, anonymised data will be deposited on the University’s open data repository. The data collected are intended to benefit the general public and to inform policy decision making; we will therefore preserve all data resulting from the study (with the exception of personal data) and make it publically available with as few restrictions as possible. The data will be given a CC-BY license which will require any future users to acknowledge the investigators in any subsequent publications arising from use of the data. We will disseminate a lay summary, explaining our findings and their importance. The findings will be disseminated via open access peer-review publication, conference presentations and press releases, and shared with a number of charities, practitioners and public health and policy organisations via presentations, briefing papers and web-based material.

### Statistical analysis

Firstly, a series of ANCOVAs will be used to test for differences between TPD1 and TPD2 scores on Time 2 perceived ratings of e-cigarette i) harm, ii) addictiveness, iii) social acceptability, iv) effectiveness, v) intentions to purchase and vi) intention to use. We will control for Time 1 measurements of the dependent variable (DV) in each analysis (thus testing differences between TPD1 and TPD2 conditions, controlling for baseline differences).

If no differences between TPD1 and TPD2 are observed, we will collapse these two conditions. The subsequent analysis will use a series of ANCOVAs with 3 between-factors Smoking status (Smoker vs. non-smoker), TPD presence (Present vs. Absent), COMP presence (Present vs. Absent). Dependent variables (DV) will consist of Time 2 perceived ratings of e-cigarette i) harm, ii) addictiveness, iii) social acceptability, iv) effectiveness, v) intention to quit, vi) intention to purchase and vii) intention to use e-cigarettes. We will control for Time 1 measurements of the DV in each analysis (thus testing for change over time, controlling for baseline differences). All main and interactive simple effects will be tested, and we also specifically plan a-priori comparisons between the TPD/no COMP conditions against i) no TPD/no COMP, ii) TPD/COMP, iii) no TPD/COMP, and COMP alone with i) no TPD/no COMP, ii) TPD + COMP, iii) TPD/no COMP. These will be conducted at each level of smoking status.

In addition, the same ANCOVAs will be repeated with the same IVs, DVs and covariates, but additionally controlling for cigarette dependence, previous e-cigarette exposure, and baseline intentions to quit. If differences between TPD1 and TPD2 are observed, we will conduct the above analyses, with TPD presence being expanded to have 3 levels (TPD1 vs. TPD2 vs. No-TPD). The a-priori comparisons will be conducted twice as described above, once with TPD being replaced by TPD1 and the second time with TPD being replaced by TPD2.

Exploratory analyses will be run on each of the variables to check for data distribution, outliers and the type of analysis required. Outliers will be considered on a case-by-case basis and discarded after verification. Analyses will be run using ‘Exclude cases pairwise’ so cases with missing data will not be included in the analysis. Demographics (age, occupation and highest qualification) may be related to perceptions of e-cigarettes as well as intentions to purchase and use e-cigarettes, and quit intentions, so we will explore the relationships between demographic variables and the aforementioned outcome variables.

## Discussion

This study aims to compare the effects of the EU-TPD e-cigarette health warnings and a comparative harm message on smokers’ and non-smokers’ perception of harms, addictiveness, social acceptability and effectiveness of e-cigarettes as smoking cessation aids either i) as they are implemented currently or ii) as implemented with an additional comparative health message. Additional aims are to evidence the potential effects of the TPD warnings on smokers’ intentions to purchase and use e-cigarettes in future quit attempts. Lastly, the potential effects of providing a comparative risk message either in addition to the TPD warnings or as a stand-alone message will be explored.

This area of research is important because smoking represents the greatest preventable cause of cancer. As reduced risk nicotine delivery products, e-cigarettes have the potential to decrease cancer prevalence if smokers are willing and able to switch. By putting such great emphasis on the potential addictiveness of e-cigarette products, TPD health warnings may deter smokers and dissuade e-cigarettes use in quit attempts. The influence of warning addictiveness labels on e-cigarettes have been demonstrated in previous studies which suggest that exposure to these warnings can both, increase harm perceptions and reduce intentions to use [35, 51]. In a between-subjects experiment, the addition of a warning label highlighting the addictiveness of e-cigarettes, led to an increase in risk perceptions and decrease in willingness to try the product whilst the same warning label on tobacco cigarettes did not have such an effect [36]; this suggests that the susceptibility vis-à-vis e-cigarettes’ abuse potential is greater than that of tobacco cigarettes. Given the public misperceptions of harm associated with e-cigarettes [16, 29], it is reasonable to posit that, the TPD health warnings are likely to further increase these misperceptions whilst decreasing intentions to purchase and use them in future quit attempts. Conversely, it has been previously shown that ‘lower risks’ warning labels on smokeless products have the ability to decrease harm perceptions [52]. Thus, in this study, we hypothesise that the addition of a comparative message (conveying the potential benefits of e-cigarette use to support cessation) will attenuate the effects of the TPD warnings in smokers. We do not have any a priori predictions regarding the effects of the comparative harm message in non-smokers. Ideally, a comparative harm message would reduce perceptions of harm in both smokers and non-smokers but increase intentions to use in smokers only.

The study presents some potential challenges. Previous research suggests that it is important that health messages are clear, comprehensible and credible to increase level of attention and likelihood of recall specifically for individuals with low literacy [5]. A pilot study is currently underway to help develop the most effective comparative message. In a two-stage process, a number of messages were created by the research team and/or extracted from the literature based on the relative risks of e-cigarettes compared to cigarettes. These were then evaluated for accuracy, clarity and persuasiveness by seven experts in the field. Friedman tests were conducted to select the top eight performing messages which will then be evaluated further in a general population sample in a pilot study (*n* = 1000). These messages will be rated on comprehensibility, credibility, and convincingness in order to increase the reach and accessibility of the comparative health message which will be used in the current study. A second potential challenge may be that the aims of the study become clear to participants. In order to minimise this, unrelated filler questions (as described above) will be added following exposure to the stimuli to distract participants from possibly guessing the aims of the study.

Health warnings, such as those implemented by the TPD, may inadvertently deter smokers from initiating use and substituting their tobacco use for e-cigarettes use if their content is deemed too negative. This may be damaging for smokers given that e-cigarettes have been suggested as useful tools to prevent relapse [33, 53]. Conversely, it is possible that our comparative harm message, particularly when delivered alone, may encourage non-smokers to use an e-cigarette. We will therefore fully debrief our participants at the end of the study by presenting accurate information about e-cigarettes, highlighting that the products are not recommended for non-smokers but constitute a less harmful alternative for smokers and that some smokers have found them helpful for quitting smoking.

We hope that our study will allow us to identify the most effective message or combination of messages that will discourage e-cigarette use among non-smokers whilst encouraging use among smokers. Findings of this study have clear relevance for the public health as they will be beneficial in furthering our understanding of how best to communicate relative health risks associated with e-cigarettes.

## Abbreviations

COMP: Comparative Health message
EU-TPD: European Union Tobacco Products Directive
FTCD: Fagerström Test for Cigarette Dependence
GDPR: General Data Protection Regulations
MTSS: Motivation to Stop Scale
ONS: Office for National Statistics
OR: Odd ratio
